# Application of Non-Viral Vectors in Drug Delivery and Gene Therapy

**DOI:** 10.3390/polym13193307

**Published:** 2021-09-28

**Authors:** Shuaikai Ren, Mengjie Wang, Chunxin Wang, Yan Wang, Changjiao Sun, Zhanghua Zeng, Haixin Cui, Xiang Zhao

**Affiliations:** Institute of Environment and Sustainable Development in Agriculture, Chinese Academy of Agricultural Sciences, Beijing 100081, China; ren19801266562@163.com (S.R.); wangmengjie@caas.cn (M.W.); wangchunxin@caas.cn (C.W.); wangyan03@caas.cn (Y.W.); sunchangjiao@caas.cn (C.S.); zengzhanghua@caas.cn (Z.Z.); cuihaixin@caas.cn (H.C.)

**Keywords:** non-viral vectors, polymers, liposomes, gold nanoparticles, mesoporous silica nanoparticles, carbon nanotubes

## Abstract

Vectors and carriers play an indispensable role in gene therapy and drug delivery. Non-viral vectors are widely developed and applied in clinical practice due to their low immunogenicity, good biocompatibility, easy synthesis and modification, and low cost of production. This review summarized a variety of non-viral vectors and carriers including polymers, liposomes, gold nanoparticles, mesoporous silica nanoparticles and carbon nanotubes from the aspects of physicochemical characteristics, synthesis methods, functional modifications, and research applications. Notably, non-viral vectors can enhance the absorption of cargos, prolong the circulation time, improve therapeutic effects, and provide targeted delivery. Additional studies focused on recent innovation of novel synthesis techniques for vector materials. We also elaborated on the problems and future research directions in the development of non-viral vectors, which provided a theoretical basis for their broad applications.

## 1. Introduction

With the development of biotechnology, drug delivery and gene therapy play an important role in the treatment of many diseases such as hereditary diseases, malignant tumors, cardiovascular diseases, infectious diseases, and neurodegenerative diseases [1,2,3,4,5,6]. However, there are some drawbacks containing rapid degradation [7,8,9], nontargeted delivery [10,11], unsatisfactory efficacy [12], multiple side effects [13,14] after nucleic acids, proteins, peptides, and other substances entering the body circulation. Therefore, suitable vectors, effective transport route, or chemical modification are necessary to improve the pharmacokinetic properties [15,16,17,18]. A growing number of vectors for gene therapy or vaccines and carriers for drug delivery have been extensively researched owing to their facile use, targeting ability, high bioavailability, and good biocompatibility [19,20,21].

**Figure 1:**
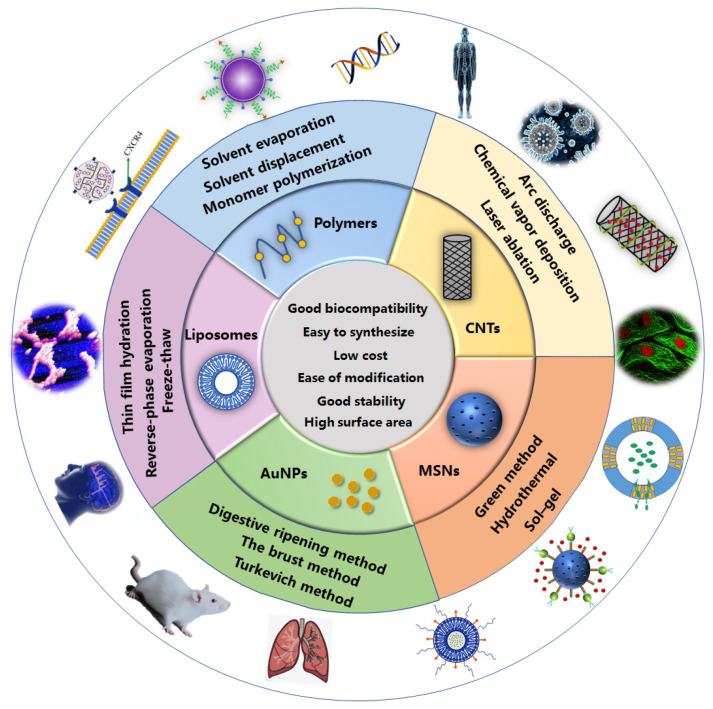
The characteristics, preparation methods, and biomedical applications of several non-viral vectors.

Viruses, such as adenovirus, vesicular stomatitis virus, cytomegalovirus, lentivirus, and retrovirus, are commonly used vectors because of highly infectious, effective delivery, and efficient expression [22,23,24,25]. However, viral vectors have several limitations including toxicity, immunogenicity, carcinogenicity, high cost, and difficulty of large-scale production in clinical practice [26,27,28]. Consequently, more and more scientists have turned their attention to the development of non-viral vectors and carriers [29,30,31]. Recent studies have shown that non-viral vectors have the following advantages: low immunogenicity, biodegradability, easy synthesis, low cost of production, and no restriction on the size of the molecules to be introduced [32,33,34,35,36]. The most extensively researched non-viral vectors are mainly polymers, liposomes, and nanoparticles [37,38,39,40,41,42]. This review introduces several non-viral vectors that have been extensively studied in the past few decades and summarizes their biomedical applications, providing a theoretical basis for the development of new non-viral vectors in the future (Figure 1). Table 1 shows the characteristics and commonly used preparation methods of several non-viral vectors. Table 2 shows the patent reports related to non-viral vectors in recent years.

## 2. Polymers

Recent trends in biodegradable polymers, especially aliphatic polymers, indicate significant developments in terms of novel design strategies and clinical biomedicine applications [58]. Polymer as a non-viral vector has the following advantages: (1) easy to synthesize and low cost [59]; (2) multiple polymers are biodegradable [60]; (3) no immunogenicity [61]; (4) allow being extensively modified [62]; (5) ability to protect the nucleic acid drugs from various enzymes by forming polyelectrolyte complexes [63]. There are four main types of production methods: solvent evaporation, emulsification–solvent diffusion, solvent displacement and monomer polymerization [59]. Various polymers such as dendrimers, polylactic acid (PLA), polyethylenimine (PEI), and chitosan (CS) have been widely used in delivery systems [51,64,65,66,67,68]. Table 3 summarizes the structural characteristics, synthesis methods and properties of several polymer materials.

### 2.1. Dendrimers

Dendrimers are linear polymers with dendron on each repeating unit and have a hyper-branched 3D structure [61,69]. Their size, degree of branching and functionality can be controlled and adjusted through the synthetic procedures [70]. Meanwhile, dendrimers contain a variety of peripheral functional groups, which can be functionally modified using surface engineering technology such as antibody, transferrin, biotin, folic acid, galactose, and peptide [71,72,73]. A variety of dendrimers such as poly (propylene imine) (PPI) dendrimers, polyamidoamine (PAMAM) dendrimers, and poly-L-lysine (PLL) dendrimers were synthesized by divergent and convergent approaches [74]. Guan et al. prepared fluorescent PAMAM dendrimer by conjugating PAMAM dendrimers to fluorescein. The vector has low cytotoxicity and high siRNA binding affinity which can improve the efficiency of Cy5-siRNA delivery in A549 cells [75]. Mastorakos et al. prepared the hydroxyl PAMAM dendrimer-based gene vectors which had high gene transfection efficiency and the stability of compound can be improved after polyethylene glycol treatment [76]. Liaw et al. prepared targeted novel hydroxyl dendrimer to deliver CSF-1R inhibitor BLZ945 (D-BLZ), these dendrimers penetrated into orthotopic brain tumors and localize specifically within TAMs. In vivo experiments on mice showed that the dendritic polymer could improve the therapeutic effect of D-BLZ on glioblastoma [77].

### 2.2. Polyethylenimine

**Figure 2:**
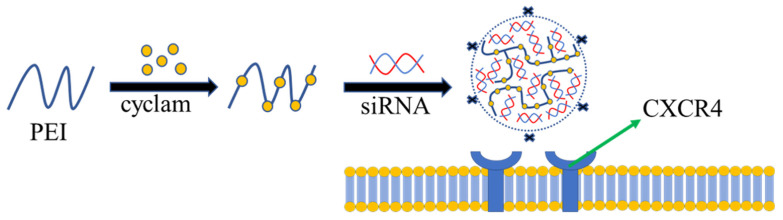
Cyclam modified PEI is used for the delivery of siRNA.

Various molecular weights of PEI can be synthesized by linear and branched forms [78]. Because PEI has a large amount of positive charge on its surface, it can be adsorbed together with negatively charged nucleic acid drugs through electrostatic action to protect them from lysosomal degradation [79,80,81,82,83]. However, PEI cannot be degraded in vivo, and its high toxicity limits its application development [84,85]. Various polyethylenimine derivatives containing coordination groups have been developed to reduce toxicity [86,87]. Mattheolabakis et al. used polyethylenimine, hyaluronic acid, and polyethylene glycol to produce a polymer with a good ability to deliver siRNA to A549 cells [88]. Zhou et al. prepared a PEI derivative modified by a cyclic amine derivative. Compared with unmodified PEI, modification with cyclic amine derivatives can significantly reduce cytotoxicity. At the same time, the polymer has a good antagonistic effect on Chemokine receptor CXCR4, and has a good inhibitory ability on tumor cell invasion (Figure 2) [83]. Low molecular weight PEI has lower toxicity, but the transfection efficiency is correspondingly lower [89]. More and more studies have been conducted to modify low molecular weight PEI to improve transfection efficiency [90,91]. Zhang et al. modified PEI 600 Da with aromatic rings in order to improve DNA affinity. Cell uptake experiments showed that the polymer had higher transfection efficiency for DNA compared with PEI 25 kDa. Meanwhile, the toxicity of the polymer has low toxicity in both 7702 and HeLa cells by CCK-8 assay [92].

### 2.3. Chitosan

Chitosan (CS) is one of the most abundant biopolymers derived from natural chitin that commonly exists in the exoskeletons of arthropods, crustacean shells, insects, and fungal cell walls [93]. CS can be degraded by internal enzymes, which makes chitosan have good biocompatibility [94,95]. Like other cationic polymers, chitosan is linked to nucleic acids by electrostatic interaction [96,97]. However, the poor solubility in water and low transfection efficiency are the main factors limiting its application [98,99,100]. The presence of amino and hydroxyl groups makes chitosan easy to modify, modification of chitosan with other substances such as PEI, gold nanoparticles, PLGA, and PEG have been widely reported [101]. Chen et al. incorporated hydrophobic deoxycholic acid (DCA) onto the chitosan backbone of poly (amidoamine) dendronized chitosan derivative (PAMAM-Cs) to obtain an amphiphilic derivative-PAMAM-Cs-DCA. Doxorubicin was wrapped inside the particle, and pDNA was electrostatically adsorbed on the surface of the particle. The system delivered both pDNA and drugs at the same time, and the transfection efficiency reached 74%. These results suggested that PAMAM-Cs-DCA NPs hold great promise to co-deliver chemotherapeutics and nucleic acid drugs [102]. Lee et al. prepared the triphenylphosphonium-glycol chitosan derivative (GME-TPP) with 36% substitution by Michael addition. GME-TPP microspheres successfully targeted DOX delivery to mitochondria in cells, which indicated the microsphere possess great potential as effective drug delivery carrier [103]. Babii et al. synthesized mannosyl chitosan with a degree of substitution of 15%. The particle has high encapsulation efficiency for CpG oligodeoxynucleotides (CpG ODN) and can target CpG ODN to immune cells, which indicated the particle may be used as an efficient carrier for intracellular CpG ODN delivery [104]. Masjedi et al. prepared targeted nanoparticles by modifying N, N, N-trimethyl chitosan with hyaluronic acid, which had low toxicity and high transfection efficiency for siRNA. The particle loaded with siRNA can block the proliferation of cancer cells by inhibiting the expression of IL-6/STAT3 [105].

### 2.4. Polylactic Acid/Poly (Lactic-Co-Glycolic Acid)

PLA and PLGA are biodegradable functional polymer organic compounds with good biocompatibility and encapsulation properties which can be metabolized in the body [106,107]. The synthesis of polylactic acid by direct condensation is described in the following four ways: (1) direct condensation polymerization; (2) azeotropic dehydration condensation; (3) lactide ring-opening polymerization; (4) double emulsion (water/oil/ water) solvent evaporation technique [108,109,110]. The characteristics of strong plasticity, low price and good versatility have enabled them to be developed for biomedical applications such as drug delivery [111,112,113]. Zabihi et al. prepared poly (lactide-co-glycerol) (PLG) particles by combining hyperbranched polyglycerol and PLA. The encapsulation efficiency of this particle on tacrolimus is 14.5%, which was able to improve the skin penetration and therapeutic efficiency of this therapeutic agent [114]. Ren et al. prepared a dextran modified PLGA microsphere that delivered IL-1 receptor antagonist (IL-1RA). The microsphere can prolong the half-life of IL-1RA, allowing it to be released continuously. The results showed that IL-1ra-loaded dextran/PLGA microsphere might be a useful tool to combat periodontal disease [115]. Bazylińskaet al. prepared effective nanocarriers coated with PLGA, PLGA-PEG, or PLGA-FA by double emulsion evaporation process, which enabled co-encapsulation of cisplatin and verteporfin. The nanocarriers successfully delivered cargo to target cells and significantly enhanced the ability of drugs to kill cancer cells [116].

### 2.5. Amino Acid Derived Biopolymers

Amino acids have become promising biomaterials for their abundant source and diverse functional groups. Various polymerization methods are used to synthesize different types of amino acid derived biopolymers such as polyamides(PA)s, polyesters(PE)s, poly(ester-amide)s(PEA)s, polyurethanes(PU)s, and poly (depsipeptide)s (PDP)s [117]. Commonly used synthesis pathways are as follows: Direct polycondensation [118]; solution or activated polycondensation [119]; ring-opening polymerization [120]; interfacial polymerization [121]; melt polycondensation [122]; chemoenzymatic synthesis [123]. Poly(α-amino acid)s have the capability of readily self-assemble into discrete, stable, structures in solution. The positive charge of poly(beta-amino ester)s can bind to nucleic acids and be internalized into cells. At the same time, they can escape from the endolysosomal compartment and release nucleic acids into the appropriate cell compartment for gene delivery through a variety of targeted degradation mechanisms [68]. In addition, abundant functional groups provide multiple modification sites for amino acid derived biopolymers. Various ligand-modified amino acid derived biopolymers were extensively studied in drug delivery (Table 4).

### 2.6. Alginates

Alginate (ALG) is a linear copolymer compound which has (1, 4)-linked-β-D- mannuronic (M) and α-L-guluronic (G) acid units [130]. The composition and length of the M and G units determine the molecular and physicochemical properties of ALG. ALG is a widely used anionic biopolymer due to its easy availability, hydrophilicity, biodegradability and versatility. The hydroxyl groups and carboxyl groups of ALG can be modified easily by oxidation, acetylation, and esterification reactions [131]. The wide particle size distribution of ALG enables it create complexes with various other biomaterials by electrostatic interactions, chemical modification, or crosslinking [132]. The most important property of alginates is their ability to form ionic gel in the presence of polyvalent cations. So–gel is the most commonly used form of carrier for ALG. In recent years, the methods of producing hydrogels included ionic crosslinking, covalent crosslinking, phase transition, cell crosslinking, free radical polymerization, and click chemistry [130]. Alginate hydrogels have outstanding properties such as high-water content, nontoxicity, soft consistency, and biodegradability [133]. Meanwhile, alginate hydrogels can regulate the release of the drug according to the pH of the surrounding medium [134]. In addition, ALG can also be developed into microspheres and nanoparticles for drug delivery. Table 5 illustrates several alginate-based drug delivery systems.

## 3. Liposomes

Liposomes are spherical vesicles composed of one or more layers of phospholipids which belong to amphiphilic molecules, hydrophilic drugs are encapsulated in a water core, and hydrophobic drugs are embedded in the lipid bilayer of the vesicle [141,142,143]. Liposomes as carriers have many advantages, including low toxicity, good biocompatibility, improved pharmacokinetics, and ease of synthesis [144,145,146]. The commonly used preparation methods are thin film hydration, reverse-phase evaporation, injection, dehydration-rehydration, and freeze-thaw. Liposomes are widely used in cancer treatment, viral infection, infectious disease, vaccines, and other medical research [147,148,149,150]. However, unmodified liposomes are unstable in structure, thus are easily eliminated in the body’s circulation, making drugs unable to effectively reach target organs and target sites [151,152,153]. Therefore, various ligand-targeting liposomes and stimulus-responding liposomes have been developed to improve the delivery and targeting performance of liposomes [154,155,156,157,158,159]. Table 6 shows that liposomes modified with different ligands to deliver different cargos.

### 3.1. Ligand-Targeting Liposomes

**Figure 3:**
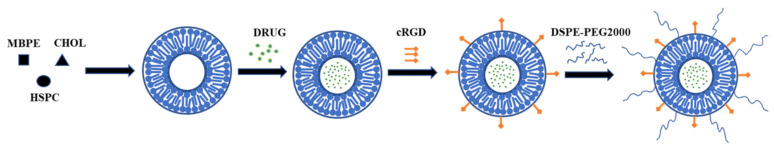
Schematic representation for preparation of RGD-DXRL-PEG.

Peptides as ligands have the advantages of small size, easy production, and high stability [170]. Peptides can be combined with liposomes through various covalent and non-covalent bonds, and are mainly divided into cell-penetrating peptides (CPP) and cell-targeting peptides (CTP) [171,172,173]. RGD sequences are the most widely used class of liposomal binding peptides, especially in tumor therapy [174]. Kang et al. developed a cyclic peptide c(RGDyC) modified liposomal delivery system to deliver the integrins αvβ3, which had a higher cellular uptake compared with liposomes without c(RGDyC) [175]. Belhadj et al. designed a Y-shaped multifunctional targeting material c(RGDyK)-pHA-PEG-DSPE to deliver DOX, which prolonged the survival time of mice [176]. The encapsulation rate of RGD-DXRL-PEG liposomes prepared by Chen et al. for doxorubicin was more than 98%, and the cellular doxorubicin uptake for RGD-DXRL-PEG was about 2.5-fold higher than that for DXRL-PEG (Figure 3) [177]. CPP typically contains 5 to 35 amino acid residues and is widely used in cancer treatment [178]. Ding et al. constructed CPP-modified pH-sensitive PEGylated liposomes (CPPL) which had high cell-penetrating and endosomal escape abilities [179]. Hayashi et al. developed H16 peptide-modified liposomes (H16-Lipo) which effectively delivered alpha-galactosidase A (GLA) to intracellular lysosomes and improved proliferation of GLA knockdown cells [160]. Some other types of peptides have also been used to modify liposomes. Chen et al. used peptide-20 modified liposome as a carrier for DOX delivery, and U87 cells had a high uptake rate of this liposome [177]. Jhaveri et al. used ferritin receptors modified liposomes to deliver resveratrol, which has a good effect on inhibiting tumor growth and improving the survival rate of mice [161]. Wei et al. developed a lactoferrin modified, polyethylene glycolated liposomes for doxorubicin delivery. The results of experiments in mice indicated that the liposome-loaded DOX has the potential to treat hepatocellular carcinoma [162].

Various immune liposomes can be obtained by attaching antibodies to the surface of liposomes using surface engineering techniques [180,181,182]. Gao et al. developed a liposome system modified with anti-EGFR Fab to deliver DOX and ribonucleotide reductase M2 siRNA, in vivo and in vitro experimental results showed that the vector system can improve the efficiency of gene therapy and had a certain therapeutic effect on hepatocellular carcinoma [183]. Saeed et al. prepared the immunoliposomes coupled to anti-MAGE A1 TCR-like single-chain antibody which can be specifically bound to and be internalized by positive melanoma cells [184]. Zang et al. prepared liposomes modified by PEG and anti-EphA10 antibody, the immunoliposome significantly improved the transfection efficiency of siRNA in MCF-7/ADR cells [163].

An aptamer is a short synthetic single stranded DNA or RNA that can specifically bind to the target through hydrogen bonds, Van der Waals forces and electrostatic interactions [185,186]. Using aptamers as ligands has the characteristics of small volume, simple synthesis process, low toxicity, good stability, high affinity, and good targeting selectivity [187]. Alshaer et al. used anti-CD44 aptamer (APT1) modified liposome as a carrier system for siRNA delivery and achieved a good gene silencing effect in tumor cells [164]. Powell et al. used Aptamer A6modified liposome as a vector to deliver siRNA to breast cancer cells which enhanced cytotoxicity and antitumor efficacy [188]. Li et al. combined Aptamer AS1411 with PEGylated liposome surface to prepare a targeted carrier for siRNA delivery. Cell uptake experiment results showed that the accumulation of siRNA in tumor cells was greater than that in normal cells. Meanwhile, the carrier system showed significant silencing activity in tumor xenograft mice and inhibited the melanoma growth which indicated that the targeted delivery system of liposomes may have potential in the treatment of melanoma [189].

Molecules such as folate and sugars also serve as ligands for liposomes [190,191,192]. There are also studies devoted to the development of liposome carriers modified with various ligands, multivalent ligands have multiple binding groups and enhance the therapeutic efficacy of drugs [193]. Kang et al. prepared a dual ligand liposome drug delivery system modified with Pep-1 peptide and folate which showed higher cellular uptake and cytotoxicity in HeLa cells as compared to chimeric-ligand oriented liposomes [194]. Zong et al. prepared a dual ligand liposome drug delivery system modified with cell-penetrating peptide (TAT) and transferrin, which effectively delivered drugs to targeted tumor cells, the results of in vivo experiments also demonstrated that this drug delivery system could improve the survival time of brain tumor-bearing animals [195].

Abbreviations: HSPC, hydrogenated soybean phosphatidylcholine; CHOL, cholesterol; MBPE, maleimidobenzoylphosphatidylethanolamine; DSPE-PEG2000, N-(carbonyl-methoxypolyethylene glycol 2000)-1, 2-distearoyl-sn-glycero-3- phosphoethanolamine sodium salt; DRUG, doxorubicin; DXRL-PEG, DXR-loaded PEGylated liposomes; RGD-DXRL-PEG, cRGD-modified DXRL-PEG.

### 3.2. Stimulus-Responding Liposomes

**Figure 4:**
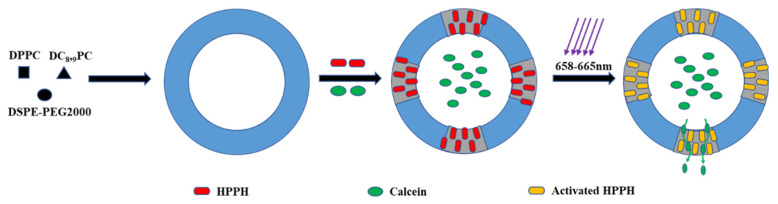
Design consideration of light-responsive liposomes.

Internal physiological conditions and external stimuli were used to promote the release of drug delivery systems in specific locations and environments to alter pharmacokinetic characteristics [196,197]. Depending on the stimulus, scientists developed various liposome drug carrying systems such as temperature-responsive liposomes, pH-responsive liposomes, ultrasound responsive liposomes, magnetic-field responsive liposomes, redox-responsive liposomes, light-responsive liposomes, and enzyme-responsive liposomes. Needham et al. prepared a kind of temperature sensitive liposome using dipalmitoylphosphatidylcholine (DPPC), monopalmitoylphosphatidylcholine (MSPC), and distearoylphosphatidylethanolamine (DSPE)-PEG2000. The liposome is relatively stable at 37 °C. When the temperature reaches 41.5 °C, 31% of the drug can be released within one to two seconds which was much higher than the unmodified liposome group [165]. Zhao et al. prepared a pH-responsive liposome drug delivery system using tumor-specific pH-responsive peptide H7K(R2)2 as a ligand. In vitro experiments proved that the drug delivery system effectively released drugs under acidic conditions, and in vivo experiments showed that the system had a good anti-tumor ability in C6 tumor-bearing mice [166]. Clares et al. used a reproducible thin film hiatus technique to prepare magnetic liposomes coated with 5-fluorouracil. Magnetic field caused the release of the drug and a good inhibition effect was observed in human colon cancer cells [167]. Sine et al. prepared a light-responsive liposome encapsulated with 2-(1-hexyloxyethyl)-2-devinyl pyropheophorbide-A and calcein, laser irradiation (660 nm, 90 mW) can promote drug release which showed enhanced antitumor efficacy (Figure 4) [198]. Chi et al. prepared redox-responsive liposomes using hyaluronic acid as a compound. The drug can be effectively released when the liposome is exposed to reduced conditions. All animals treated with liposomal formulations survived in contrast to those animals treated with free-DOX, indicating the liposomal formulation have an effective tumor suppressive effect [168]. Song et al. synthesized enzymatic-responsive liposomes using the enzymatically cleavable peptide linkers GFLG (Gly-Phe-Leu-Gly) as the ligand system. After GFLG was degraded by endo-lysosomal enzyme, the encapsulated pDNA was released and the transfection efficiency was 100 times higher than that of the control group without GFPG modification [169].

Abbreviations: DPPC, 1,2-dipalmitoyl-sn-glycero-3-phosphocholine; DC_8_,_9_PC, 1,2 bis(tricosa-10,12-diynoyl)-sn-glycero-3-phosphocholine; DSPE-PEG2000, 1,2-distearoyl -sn-Glycero-3-Phosphoethanolamine-N-[Methoxy(Polyethylene glycol)-2000].

## 4. Gold Nanoparticles

**Figure 5:**
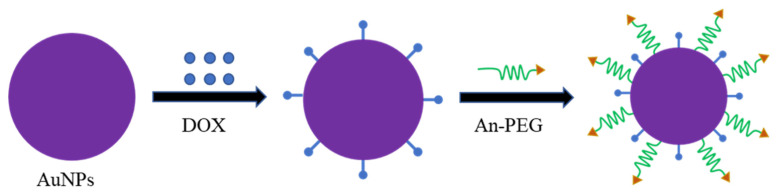
An-PEG modified gold nanoparticles are used to deliver DOX. Abbreviations: DOX, doxorubicin; An, Angiopep-2.

Gold nanoparticles (AuNPs) have good stability and biocompatibility [199]. Quantum size effect and high surface area-to-volume ratio make AuNPs have high drug loading [200]. Meanwhile, gold nanoparticles are easy to modify and can improve the pharmacokinetics of many drugs which makes gold nanoparticles widely used in immune analysis, drug delivery, and detection of cancer cells and microorganisms [201,202,203]. For example, Ruan et al. synthesized the Angiopep-2-PEG modified AuNPs which could specifically deliver and release DOX in glioma and significantly expand the median survival time of glioma-bearing mice (Figure 5) [204]. The synthesis methods of gold nanoparticles include chemical synthesis and biological synthesis. The commonly used chemical methods include the turkevich method, the brust method, and digestive ripening method [205,206,207]. The chemosynthesis method has some limitations including low yield, difficulty in controlling particle shape, strict preparation conditions, and poor biocompatibility [208,209,210,211]. Therefore, more and more scientists are using friendly biosynthesis methods to synthesize gold nanoparticles.

Bacteria are important biological sources for the synthesis of AuNPs. The extracellular enzymes work as a reducing agent in the reduction of metals during the synthesis of microbial NPs and NADH-dependent reductase can carry out electron transfer from NADH, leading to reduction of metal ions [212,213]. Parastoo et al. prepared the gold nanoparticles with spherical, hexagonal, and octagonal shapes by reducing HAuCl_4_ in supernatant microbial of bacillus cereus culture [214]. Sharma et al. screened a marine bacterium from different sea cost in India to produce gold nanoparticles. The prepared gold nanoparticles were mostly spherical with a particle size of 10 nm [215]. Fungi also can be used to synthesize gold nanoparticles. Sanghi et al. synthesized intracellular gold nanoparticles with Phanerochaete chrysosporium and demonstrated that ligninase played an important role [216]. Molnár et al. synthesized gold nanoparticles of different sizes (between 6 nm and 40 nm) under controlled experimental conditions [217].

As a cheap biological material, plants were used to synthesize gold nanoparticles in recent years. Different plant species, different parts of the same plant species such as leaves, roots, stems, and fruits can be used as raw materials for the synthesis of gold nanoparticles [218]. Gopinath et al. synthesized spherical gold nanoparticles with particle size of 20 nm to 50 nm by aqueous leaf extract of terminalia arjuna [219]. Yu et al. used Citrus Maxima (C. Maxima) fruit extract to synthesize gold nanoparticles with an average particle size of 25.7 nm [220]. In addition, some studies have shown that gold nanoparticles can be synthesized from seaweed [221,222]. Table 7 shows the various biomaterials that can be used to synthesize gold nanoparticles.

The size and shape of gold nanoparticles can be tuned by controlling the synthesize conditions such as temperature, type of surfactant, and concentration of metal matrix in both chemical and biosynthetic methods [238]. The size and shape of gold nanoparticles strongly influence their toxicity, drug loading, and penetration properties, and then affect their biomedical applications. A study showed that 5 nm AuNPs in a concentration of more than 50 μM were associated with cytotoxic effects, while 15 nm AuNPs presented good biocompatibility [239]. Karol et al. studied the relationship between toxicity and shape of gold nanoparticles (rods, stars, and spheres). The results showed that star shape gold nanoparticles has the highest anticancer potential but has the slowest cellular uptake due to their big size, while the sphere shape gold nanoparticles exhibited the most safety, the fastest cellular uptake and weak anticancer potential [240]. A study about the size dependence of the antiviral activity of AuNPs demonstrated that small particles (2 nm) had no inhibitory effect for influenza virus, while medium-sized AuNPs (14 nm) inhibited the virus binding and infection [241].

## 5. Mesoporous Silica Nanoparticles

In 1992, the first ordered mesoporous silica (MCM type) was synthesized by the Mobile Research and Development Corporation [242,243]. Subsequently, many other types of mesoporous silica nanomaterials (MSNs) such as BSA type, HMM type, KIT type, KCC type, FSM type, and TUD type were synthesized using a variety of improved methods. Table 8 shows the specific example of the synthesis of various MSNs. Various distinctive properties of MSNs including substantial surface area, large pore size, low density, good adsorption and encapsulation capacity, controllable superficial charge, ease of modification, and high biocompatibility showed great potential in drug delivery applications [244,245,246,247,248,249]. The synthesis techniques of MSNs can be classified into sol–gel, as well as hydrothermal and green method (Table 9) [250].

Regardless of the synthesis method, studies have shown that selection of surfactant molecule, silica precursors, solvents, reaction temperature, stir speed, and pH of the media affect the shape, size, surface area, and pore size of MSNs [263,264], and these physical properties further affect the drug loading, toxicity, and uptake efficiency of the carriers [265,266,267]. Cho et al. found that compared with MSNs with a particle size of 100 nm or 200 nm, MSNs with a particle size of 50 nm had the fastest clearance rate in urine and bile [268]. Lu et al. prepared a series of MSNs with particle sizes of 30 nm, 50 nm, 110 nm, 170 nm, and 280 nm, the cellular uptake amount of 50 nm nanoparticles was much higher than other groups [269]. In addition, studies showed that rod-shaped MSNs internalize faster and higher on tumor cells than spherical MSNs [270]. Meanwhile, the pores of MSNS have a large surface area, and for different drugs, the release of drugs can be controlled by regulating the size of the pores [271]. Mellaerts et al. prepared four SBA-15 MSNs with pore size varying from 4.5 to 9.0 nm, and they found that the increase of the pore size from 4.5 to 6.4 nm significantly improved the release of itraconazole, while a further increase to 7.9 and 9.0 nm revealed a slight improvement in the release profile [272].

**Figure 6:**
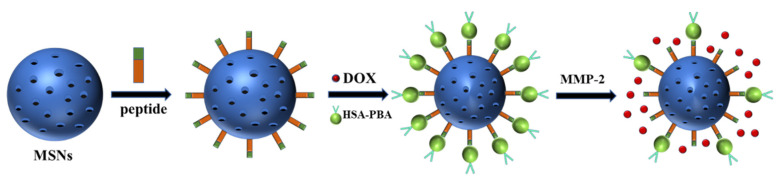
Design consideration of enzyme-responsive MSNs. Abbreviations: DOX, doxorubicin; HAS, human serum albumin; PBA, phenylboronic acid; MMP-2, matrix metalloproteinase-2.

However, two challenges of MSNs may limit its broader application. The open pores of MSNs are ideal reservoirs for drugs, which adversely trigger a premature release of drugs before reach the target [266]. A simple way to minimize the leakage is the attachment of the drugs through a cleavable bond onto the inner surface of the particle [273]. Wong et al. connected doxorubicin (DOX) and zinc(II) phthalocyanine (ZnPc) to form a DOX-ZnPC complex using an acid cleavable hydrazone linker, and the resulted delivery system achieved drug release under acidic conditions [274]. Another method involved loading one drug inside the pores and attaching another drug at the outlet of the pores [273]. Willner et al. loaded the anticancer drug mitoxantrone into boric acid modified MSNs, the pores were capped with gossypol, then the capping units unlocked the pores and the drug is released under mild acidic conditions [275]. Another challenge is that unmodified MSNs lack the active targeting and slow-release ability; therefore, various responsive delivery systems were prepared through surface modification. Various ligands such as polyethylene glycol, folic acid, polyethylenimine, hyaluronic acid, phenyl, thiol, and sulfonate have been reported to modify MSNs [276,277,278,279,280]. After ligand modification, MSNs can realize the function of drug release under specific environment including pH, redox, enzyme, temperature, magnetic field, and light stimulation. Liu et al. designed and fabricated a biocompatible, enzyme-responsive drug delivery system based on MSNs for targeted drug delivery in vitro and in vivo. The system demonstrated sensitivity to MMP-2 for drug delivery, leading to cell apoptosis which displayed a good curative effect on the inhibition of tumor growth with minimal toxic side effects (Figure 6) [281]. Table 10 shows various stimulus-responsive-MSNs for controlled release.

## 6. Carbon Nanotubes

The diameter of CNTs is in the order of nano and the length is in the order of micron, giving them a high aspect ratio and large surface area [293,294,295,296]. Due to their outstanding properties such as good adsorption ability, excellent chemical stability, high tensile strength, significant electrical, and thermal conductivity, CNTs have been used in a variety of biomedical fields, especially drug delivery and cancer treatment [297,298,299]. There are three main ways to manufacture CNTs, including arc discharge, chemical vapor deposition (CVD), and laser ablation [300]. Toxicity is often a concern in clinic applications. Several physical and chemical factors including purity of the material, morphology, and administration route are crucial for the toxicity of CNTs [301]. It has been reported that residual transition metal catalysts such as iron, cobalt or nickel contained in the pristine CNTs can catalyze the intracellular formation of free radicals and oxidative stress leading to cytotoxic effects [302]. Therefore, the purification of CNTs by exposing them to high temperatures or bathing sonication assisted acid oxidation reduced the remains of catalytic metals used in their synthesis, increasing their biocompatibility and decreasing the toxicity levels [303]. In addition, the modification of CNTs is also an effective method to reduce their toxicity [304].

**Figure 7:**
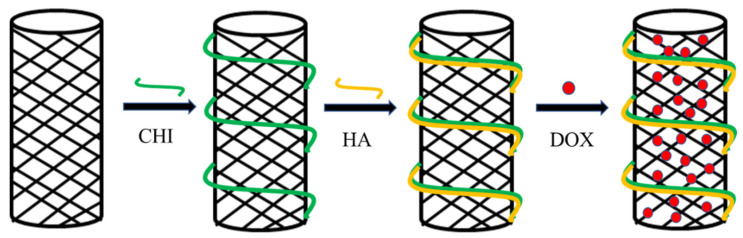
CHI- and HA-modified SWCNTs were used to deliver DOX. Abbreviations: CHI, chitosan; HA, hyaluronan; DOX, doxorubicin.

CNTs tend to agglomerate uncontrollably due to Van der Waals forces among bundles and high surface energy, which hinders their dispersion in almost all organic and inorganic solvents [298]. Meanwhile, the morphology and chemical properties of CNTs are the main factors affecting their entry into target cells [305]. Chemical functionalization can modify CNTs’ electronic properties, reduce agglomeration, and improve their solubility in different solvents [306]. The main approaches for CNTs’ functionalization can be divided into two main groups including covalent functionalization and non-covalent functionalization. The covalent functionalization mainly relies on covalent bond to connect carbon nanotubes to molecules. The non-covalent modification mainly relies on Van der Waals forces and electrostatic interaction to connect carbon nanotubes to molecules [307]. Antibodies, peptides, hyaluronic acid, oligonucleotides, polyethylene glycol, and other substances are often used to modify CNTs [308,309]. Mo et al. prepared a pH-responsive drug delivery system with SWCNTs as the core and CHI and HA as ligands (Figure 7) [310]. Table 11 provides detailed cases of various functionalized CNTs delivered to different cargoes.

## 7. Conclusions and Perspectives

Viral vectors are the earliest and most widely used type of vectors. However, toxicity, immunogenicity, carcinogenicity, high cost, and other issues limit their broader application. The investigation of non-viral vectors such as f liposomes, polymer, gold nanoparticles, mesoporous silica nanoparticles, and carbon nanotubes in medical research is growing rapidly. In this contribution, the application of non-viral vectors in drug delivery and gene therapy is summarized. Non-viral vectors can prevent the premature degradation of nucleic acids, proteins or drugs, prolong therapeutic effect, and reduce side effects. In addition, ligand modifications make the vectors better connect with the cargo or with the target site of action, increase the loading capacity and uptake rate, as well as improve the sustained release and targeting properties of the delivery system. Polyethylene glycol, folic acid, hyaluronic acid, peptides, oligonucleotide sequences, and other ligands have been reported to modify various materials. Further research will be necessary to introduce new ligands and develop novel smart delivery systems. Furthermore, biomedical applications have high requirements for the physicochemical properties of the vectors, thus synthesizing and purifying vector materials with suitable particle size, uniform morphology, and good biocompatibility are essential. Meanwhile, the residual toxic effects of catalysts, solvents, and other substances in a synthesis process cannot be ignored. Consequently, non-viral vector materials are constantly improving new synthetic methods especially green synthesis methods, which is also a key direction of future research.

Although many studies have pointed out that non-viral vectors are biocompatible, most of the results focus on the short-term toxicity in vivo, and the protocols used in some toxicity tests are not standardized, posing an important safety concern in clinical application. Therefore, standardizing the toxicological tests and determining the safe exposure limits are crucial. Despite these challenges, with the development of novel materials and new synthetic strategies, non-viral vectors are expected to be widely applied to enhance the performance of drug delivery and gene therapy in the near future.

## Figures and Tables

**Table 1:** The characteristics and preparation methods of several non-viral vectors.

Vector	Characteristics	Preparation Methods
Polymers	Easy to synthesize Low cost Biodegradable No immunogenicity Allow to be extensively modified	Solvent evaporation Emulsification–solvent diffusion Solvent displacement Monomer polymerization Double emulsion solvent evaporation
Liposomes	Low toxicity Good biocompatibility Improved pharmacokinetics Ease of synthesis	Thin film hydration Reverse-phase evaporation Injection Dehydration-rehydration Freeze-thaw
Gold nanoparticles	Good stability and biocompatibility High surface area-to-volume ratio Easy to modify	Turkevich method The brust method Digestive ripening method Green method
Mesoporous silica nanoparticles	Substa Ntial surface area Large pore size Low density Adsorption capacity Tunable pore size Ease of modification High biocompatibility	Sol–gel Hydrothermal Green method
Carbon nanotubes	Good adsorption ability Excellent chemical stability High tensile strength Significant electrical Thermal conductivity	Arc discharge Chemical vapor deposition (CVD) Laser ablation

**Table 2:** The patent reports related to non-viral vectors in recent years.

Vector	Summary	References
Polymer	Gene transfer composition using a tri-block polymer electrolyte being polyethyleneimine-polylactic-acid-polyethylene-glycol	[43]
Polymer	A methoxypolyethylene glycol-polylactic acid block copolymer was prepared to improve the drug encapsulation rate	[44]
Polymer	The chitosan modified with a carboxymethyl group and a hexanoyl group can be used as a material for a drug carrier	[45]
Polymer	Chitosan microspheres capable of precisely controlling the release of the drug	[46]
Polymer	Alginate extraction method	[47]
Polymer	Injectable hybrid alginate hydrogels	[48]
Liposomes	A method for preparing a Decoy nucleic acid cationic liposome carrier	[49]
Liposomes	An efficient, stable human lung tissues-active targeting immune nanoliposome, with specific active lung targeting	[50]
Liposomes	A liposome preparation, a preparation method and an application thereof in treatment for related diseases caused by abnormal expression of gene	[51]
Gold nanoparticles	A method for producing confeito-like gold nanoparticles using hydroxyl peroxide in an aqueous alkaline condition in the presence of a biocompatible protecting agent	[52]
Gold nanoparticles	Method for the size controlled preparation of these monodisperse carboxylate functionalized gold nanoparticles	[53]
Silica nanoparticles	Mesoporous silica nanoparticles and supported lipid bi-layer nanoparticles for biomedical applications	[54]
Silica nanoparticles	Mesoporous silica nanoparticles with lipid bilayer coating for cargo delivery	[55]
Carbon nanotubes	Payload molecule delivery using functionalized discrete carbon nanotubes	[56]
Carbon nanotubes	Carbon nanotubes for imaging and drug delivery	[57]

**Table 3:** The information of several polymer materials.

Polymer	Structure	Synthesis Methods	Characteristics	Limitations
Dendrimers	Linear polymers with dendron on each repeating unit	Divergent approaches, Convergent approaches	Uniform size, High degree of branching, Polyvalency, Water solubility, Available internal cavities	-
Polyethylenimine	Cationic polymer of ethylenediamine monomers	-	High transfection efficiency	High toxicity
Chitosan	Repeating β -(1,4)-2-amino-D-glucose and β-(1,4)-2-acetamido-D-glucose units	Chemical method, enzymatic	Good biocompatibility	Poor solubility in water, Low transfection efficiency
Polylactic acid	The polymerization of lactic acid	Direct condensation polymerization, Azeotropic dehydration condensation, Lactide ring-opening polymerization, Double emulsion solvent evaporation technique	Strong plasticity, Low price, Good versatility	Poor hydrophilicity
Amino acid derived biopolymers	Amino acid polymerization	Direct polycondensation, Solution or activated polycondensation, Ring-opening polymerization, Interfacial polymerization, Melt polycondensation, Chemoen-zymatic synthesis	Wide-range of functional groups, Good biocompatibility	Production of by-products in the synthesis process
Alginate	Linear copolymer	Ionic crosslinking, Covalent crosslinking, Phase transition, Cell crosslinking, Free radical polymerization, Click chemistry	easy availability, hydrophilicity, biodegradability, versatility	Aggregation tendency with protein at high pHs

**Table 4:** Various responsive Amino acid derived biopolymers are used to deliver cargos.

Type	Ligands	Stimulus	Cargo	References
ssPBAE	HA	PH/redox	DOX/CXB	[124]
LPAE	-	Light	DNA	[125]
PBAE	PEG	PH	VP	[126]
PBLG	PEG	PH/Temperature	DOX	[127]
PBAE	-	PH	ATRA	[128]
SCA-PAE	HA	PH	siRNA	[129]

**Table 5:** Various alginate-based vehicles used in drug delivery.

Carriers	Type	Cargo	References
ALG/Keratin	Hydrogels	Doxorubicin	[135]
ALG/HA/Folate	Hydrogels	OXA	[136]
ALG/CS/BSA	Microcapsule	DOX	[137]
ALG/PEG	Microspheres	Polystyrene	[138]
ALG/CS	Nanoparticles	Cur	[139]
ALG/Laponite	Nanohybrids	DOX	[140]

**Table 6:** Various ligands modified liposomes to deliver different cargos.

Ligands	Stimulus	Cargo	References
H16 peptide	-	Alpha-galactosidase A	[160]
Ferritin receptors	-	Resveratrol	[161]
Lactoferrin	-	Doxorubicin	[162]
PEG and anti-EphA10 antibody	-	siRNA	[163]
Anti-CD44 aptamer	-	siRNA	[164]
DSPE–PEG-2000	Temperature	Doxorubicin	[165]
Peptide H7K(R2)2	PH	dDoxorubicin	[166]
Superparamagnetic magnetite	Magnetic Field	5-fluorouracil	[167]
Hyaluronic acid	Redox	Doxorubicin	[168]
Enzymatically cleavable peptide linkers GFLG	enzyme	pDNA	[169]

**Table 7:** Characteristics of gold nanoparticles synthesized from different raw materials.

Name of Organism	Size (nm)	Shape	References
**Bacteria**			
*Bacillus cereus*	20–50	Spherical, hexagonal, octagonal	[214]
Brevibacterium casei	10–50	Spherical	[223]
Vibrio alginolyticus	50–100	Irregular	[224]
Paracoccus haeundaensis BC74171(T)	20.93 ± 3.46	Spherical	[225]
**Fungi**			
Macrophomina phaseolina	14–16	Spherical	[226]
Morchella esculenta	16.51	Spherical and hexagonal	[227]
endophytic Cladosporium species	5–10	Spherical	[228]
Ttichoderma sp. WL-Go	1–24	Spherical and pseudo-spherical	[229]
**Plants**			
Annona muricata	25.5	Spherical	[230]
Benincasa hispida	22.18 ± 2	Spherical	[231]
Capsicum annuum	19.97	Spherical	[232]
Turnera diffusa	24	Spherical	[233]
**Algae**			
Sargassum serratifolium	5.22	slightly spherical, triangles, pentagons, and narrow square	[234]
marine red algaAcanthophora spiciferaby	<20	Spherical	[235]
marine brown algae S. ilicifolium	20–25	Near-spherical	[236]
Chlorella sorokiniana Shihira & R.W	5–15	Spherical	[237]

**Table 8:** Synthesis of different series of MSNs.

Type	Silica Sources	Surfactant	References
MCM	Sodium silicate, Tetramethylammonium silicate, Tetraethyl orthosilicate	Quaternary ammonium surfactant	[242]
BSA	Sodium silicate	C_18_TMACl	[251]
HMM	1,2-bis(trimethoxysilyl)ethane	C_18_H_37_N(CH3)_3_Cl	[252]
KIT	Tetraethyl orthosilicate, Carboxyethylsilanetriol sodium salt	Pluronic F127	[253]
KCC	Tetraethyl orthosilicate	Cetylpyridinium bromide	[254]
FSM	Layered polysilicate kanemite	Quaternary ammonium surfactant	[255]
TUD	Tetraethyl orthosilicate	Tetraethyl ammonium hydroxide	[256]

**Table 9:** Three different synthesis methods of MSNs.

Method	Silica Sources	Surfactant	Catalyst	References
Sol–gel	Sodium silicate	Polyethylene glycol	Acetic acid	[257]
	Tetrethylorthosilicate	Cetyltrimethylammonium chloride	Triethanolamine	[258]
Hydrothermal	Tetrethylorthosilicate	Cetyltrimethylammonium bromide	Ammonia	[259]
	Tetrethylorthosilicate	Pluronic F-127	Chloride acid	[260]
Green	Banana Peel	Cetyltrimethylammonium bromide	NaOH	[261]
	Tetraethyl orthosilicate	C16-L-histidine, C16-L-poline and C16-L-tryptophan	HCl	[262]

**Table 10:** Various responsive MSNs are used to deliver cargos.

Ligands	Stimulus	Cargo	References
FA-PEG-COOH	Redox	Doxorubicin and Bcl-2	[282]
Disulfide bonds modifiedpolyethylene glycol	Redox	Rhodamine B	[283]
Galactose-modified trimethyl chitosan-cysteine	PH	Doxorubicin and vascular endothelial growth factor siRNA	[284]
Succinylated ε-polylysine	PH	Prednisolone	[285]
Peptide LVPRGSGGLVPRGSGGLVPRGSK-pentanoic acid (P)	Enzyme	Anticoagulant drug	[286]
Phenylboronic acid-human serum albumin	Enzyme	Doxorubicin	[281]
Superparamagnetic magnetite nanocrystal clusters	Magnetic Field	Small interfering RNA	[287]
PEI-Iron oxide	Magnetic Field	siRNA-PLK1	[288]
PEO-b-poly (N-isopropylacrylamide) based copolymeric micelles	Temperature	Ibuprofen	[289]
Poly(N-isopropylacrylamide)-co-(1-butyl-3-vinyl imidazolium bromide) (p-NIBIm)	Temperature	Cytochrome C	[290]
1-tetradecanol	Light	Doxorubicin	[291]
Ruthenium complex	Light	Safranin O	[292]

**Table 11:** Various ligand-modified SWNTs and WWNTs are used to deliver cargos.

Type	Ligands	Cargo	Stimulus	References
SWCNTs	Polysaccharide chitosan-hyaluronic acid	Doxorubicin	pH	[310]
	Oligonucleotides	DNA/RNA	-	[311]
	Chitosan	Curcumin	pH	[312]
	Polyethylenimine with betaine	Survivin siRNA, Doxorubicin	pH	[313]
MWCNTs	Folic acid	Doxorubicin	Magnetic Field	[314]
	1-octadecanethiol-f-GNPs	Cisplatin	-	[315]
	Chitosan	Methotrexate	pH	[316]
	Distearyl phosphatidyl ethanolamine-PEG	-	Light	[317]

## Data Availability

The data presented in this study are available on request from the corresponding author.
